# Effect of Long-Term Continuous Light Exposure and Western Diet on Adropin Expression, Lipid Metabolism, and Energy Homeostasis in Rats

**DOI:** 10.3390/biology10050413

**Published:** 2021-05-07

**Authors:** Mahmoud Mustafa Ali Abulmeaty, Ali Madi Almajwal, Khalid S. Alnumair, Suhail Razak, Mai Mohammed Hasan, Amal Fawzy, Abdullah Ibrahim Farraj, Manal Abudawood, Ghadeer S. Aljuraiban

**Affiliations:** 1 Department of Community Health Sciences, College of Applied Medical Sciences, King Saud University, Riyadh 11362, Saudi Arabia; aalmajwal@ksu.edu.sa (A.M.A.); alnumair@ksu.edu.sa (K.S.A.); smarazi@ksu.edu.sa (S.R.); galjuraiban@ksu.edu.sa (G.S.A.); 2 Department of Medical Physiology, School of Medicine, Zagazig University, Zagazig 44519, Egypt; mmjewefel@zu.edu.eg; 3 Department of Medical Biochemistry, School of Medicine, Zagazig University, Zagazig 44519, Egypt; AFhassan@medicine.zu.edu.eg; 4 Department of Clinical Laboratory Sciences, College of Applied Medical Sciences, King Saud University, Riyadh 11362, Saudi Arabia; afarraj@ksu.edu.sa (A.I.F.); mabudawood@ksu.edu.sa (M.A.)

**Keywords:** Adropin, continuous light, Western diet, RORα, Rev-erb-α, energy expenditure

## Abstract

**Simple Summary:**

Behavioral characteristics of living organisms may affect the metabolism and its underlying molecular basis. The lifestyles of some modern communities include prolonged light exposure at night, and a high-fat/high-sugar-containing diet is frequently investigated. The molecular mechanisms of this unhealthy behavior might involve Adropin and some related nuclear receptors. This study examines the effect of long-term continuous light exposure and high fat/sucrose (HFS) diet on Adropin expression, RORα, Rev-erb-α nuclear receptors, key enzymes of lipid metabolism, and energy homeostasis in a rat model. The results of this study demonstrate the deleterious effects of this modern behavior on the metabolism, histology of many organs and general health. In conclusion CL and WD produced widespread derangements of energy homeostasis and lipid metabolism. The pathogenesis of this disturbance includes, at least in part, the Adropin hormone with the involvement of the RORα and Rev-erb-α nuclear receptors. Future therapeutic potential may involve Adropin.

**Abstract:**

Long-term continuous light exposure (CL) and western diet (WD) effects on Adropin expression, RORα, and Rev-erb-α nuclear receptors and energy homeostasis were studied in rats. Thirty-two male Wistar rats (250–290 g) were enrolled for 3 months in the following groups (*n* = 8/group): (a) Normal control group (NC), (b) CL group, (c) WD group, and (d) CL + WD group. Then, indirect calorimetry and food intake (FI) were measured. Finally, Adropin, hormone-sensitive lipase (HSL), adipocyte triglyceride lipase (ATGL), and free fatty acids (FFA) were measured. Additionally, the histopathology and gene expression of Enho, RORα, and Rev-erb-α genes were done. The CL alone elevated the Adropin plasma level and gene expression, increased RORα expression, and decreased the Rev-erb-α nuclear receptor expression mainly in the liver and kidney. Besides, CL increased the total energy expenditure (TEE) and decreased the respiratory quotient. WD alone or in combination with the CL reversed gene expression of Enho, RORα, and Rev-erb-α. Combined CL and WD increased the TEE, reduced the food intake, increased the ATGL, and reduced the Adropin level in addition to widespread degenerative changes in the liver, spleen, and renal tissues. The deleterious effects of CL and WD on energy homeostasis may include Adropin with the involvement of the RORα and Rev-erb-α nuclear receptors.

## 1. Introduction

The chronobiology is highly sensitive to changes in the light/dark cycle [1]. Light stimulation of the retina via activation of certain neurons in the retinohypothalamic tract could modulate the central circadian clock in the suprachiasmatic nuclei (SCN) of the hypothalamus [2]. Therefore, disturbing the light/dark cycle is involved in the disturbance of the normal chronobiologic rhythms of many regulators of the metabolic system, resulting in various disorders such as insulin insensitivity, obesity, type 2 diabetes, and the risk of cardiovascular disease [3]. Regardless of sleeping, nightshift workers or even the general population of modern communities, especially Arabic societies with high socioeconomic status, are extensively exposed to light at the night, with the expected metabolic derangements [4]. Furthermore, the modern lifestyle encounters the ingestion of higher percentages of fatty foods and beverages with added sugars, creating a complex metabolic challenge. The extensive controversy is present regarding the effect of continuous light (CL) exposure on the body weight and food intake. Previous reports that investigated the long-term CL exposure in rats mentioned a decrease in body weight gain [5], increase in body mass [6], or no change in body weight [1,7,8,9]. Food intake during CL or dim light exposures showed a similar controversy [10]. The light/dark cycle can also affect the energy expenditure by modulation of the SCN and consequent effects on the peripheral clocks in the liver, intestine, and pancreas via autonomic signals and the release of hormones [11].

Several reports stated an emerging role of Adropin as a new player in chronobiology [12]. Adropin is encoded by the Energy Homeostasis Association (ENHO) gene, which is expressed in many tissues and implicated in energy homeostasis, glucose, fatty acid metabolism, angiogenesis, and apoptosis [13]. Adropin may serve as a link between the biological clock and nutrients metabolism [14]. The circadian rhythm regulates peripheral biological clocks that time the food intake, gastrointestinal (GIT) functions, GIT hormones, hepatic metabolic pathways, and nuclear receptors in the hepatocytes [15]. Among the nuclear receptors, RORα/γ (retinoic acid receptor-related orphan receptor) and Rev-Erb-α (nuclear receptor subfamily 1, group D, member 1) may combine Adropin with the peripheral circadian rhythm of lipid and glucose metabolism [14]. The Adropin expression shows also a circadian rhythm with a peak during high feeding in the dark which is associated with activation by the RORα/γ nuclear factors [16]. Furthermore, ROR and Rev-erb-α mediate rhythms in the gene expression of the hormones controlling the lipid and carbohydrate metabolism [17]. The physiological functions of adipose tissue including lipolysis are also controlled by the central and tissue biological clocks, which coordinate the energy homeostasis and the associated metabolic paths [18].

Considering the above data, little is known about the effects of the modern lifestyle with extended periods of light exposure and consumption of high fat and added sugars on the Adropin system and the coupled nuclear receptor. Therefore, this study aimed to investigate the effect of long-term continuous light exposure and a Western diet consisting of high fat/added sucrose (WD) on the Adropin expression, expression of RORα, Rev-erb-α nuclear receptors, key enzymes of lipid metabolism, and energy homeostasis in a rat model.

## 2. Materials and Methods

### 2.1. Animals and Procedures

In total, thirty-two male Wistar rats (250–290 g, 8–10 weeks old) were obtained from the animal house of College of Pharmacy, King Saud University. Rats were randomly enrolled in one out of four groups (*n* = 8/group) for 3 months of intervention. The groups included: (a) Normal control group (NC) with normal light/dark cycle, where the light was switched on at 6 a.m. and turned off at 6 p.m., and also fed a balanced diet (complex carbohydrate 55%, fat 30%, and protein 15%); (b) Continuous light (CL) group, in which rats were also fed the balanced diet (the same as that of the NC group) and exposed to continued light exposure for three months [5]; (c) Western diet (WD) group, where butter was added to the solid food (40% fat, 45% carbohydrate, and 15% protein) with sucrose dissolved in the drinking water (34% g sucrose dissolved in the drinking water) [19]; and (d) Continuous light + Western diet (CL + WD) group, where both procedures were followed for three months. Initial body weight and weekly updates were recorded in grams (g). The basal and final body weights were used for the analysis, in addition to the amount of weight gain (final weight—basal weight). The study protocol was approved by the ethics committee in the College of Applied Medical Sciences, King Saud University under reference number CAMS 084-3839.

### 2.2. Indirect Calorimetry

All rats were individually housed in Calo-cages of the PhenoMaster system (TSE, Bad Homburg, Germany) at a normal room temperature (25 °C). The first 6 h of measurement were deleted for compensation of acclimatization bias [20]. For three consequent days, automatic measurement of volumes of respiratory oxygen (VO_2_), volumes of carbon dioxide (VCO_2_), and total energy expenditure (TEE) per hour per Kilogram (kg) of rat body weights per kg of lean body mass (0.75% of body weight) and per rat weights were done. Besides, the respiratory exchange ratio (RQ), automatic food intake (FI), and manual water intake were recorded [21]. During the 3-day stay in the phenomaster system, the light and dietary interventions were maintained according to the original group.

### 2.3. Blood Sampling

**Figure 1:**
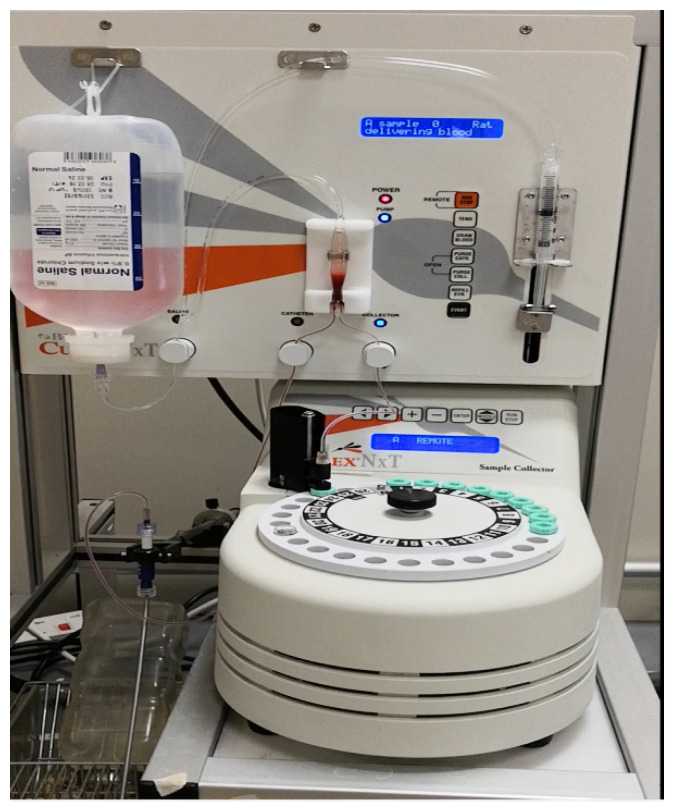
Automated blood sampling from freely mobile rats.

After indirect calorimetry, 3 rats from each group were randomly selected and operated to insert a carotid catheter [22] under general anesthesia by using a ketamine (80 mg/kg) and xylazine (12 mg/kg) mixture via the intraperitoneal route [23]. Then, a catheter was connected to a harness fitted around the neck and forelegs of the rat. The harness was linked to the Culex ABC tether and swivel system (BASi Co., West Lafayette, IN 47906, USA) which automatically withdraws blood samples from freely mobile rats (Figure 1). Serial blood samples (150 μL/each) were withdrawn at 4 time points (4, 8, 12, 16, 20, and 24 h). Blood samples were collected and centrifuged; then, the plasma was kept frozen until the time of analysis. Later on, all rats were euthanized after overnight fasting to withdraw about 5–7 mL of blood via cardiac puncture. The blood was collected in Heparinized collection tubes and centrifuged; then, the plasma samples were stored at −80 °C until the time of analysis.

### 2.4. Tissue Samples Preparation

Immediately after the cardiac puncture, tissue samples from the liver, kidney, and spleen were collected in blank tubes and frozen immediately in liquid nitrogen and stored at −80°C for gene expression analysis, and another set of tissue sample were fixed in 10% neutral formalin solution for the pathology examination. Then, pathology samples were preserved in 70% ethanol and dehydrated with ascending grades of ethanol. Then, it was cleared with xylene and embedded in paraffin wax to get paraffin blocks. Paraffin blocks were cut to obtain serial sections of 5 μm; then, they were put on the top of glass slides and stained with hematoxylin and eosin (H&E) staining. They were examined under a light microscope equipped with a high-resolution digital camera (×200 and ×400). Three rats from each group were used for pathology, and for each rat, two slides from each organ were stained. The regions of interest (ROIs) were randomly selected and viewed under the microscope. Digital images of histological sections were analyzed using image J software [24], (Image J, National Institutes of Health, Bethesda, MD, USA). Image J tools were used to measure the main pathognomonic features in the studied organs.

### 2.5. Gene Expression Analysis

The samples were transferred under the proper freezing condition to the central lab of Zagazig University, School of Medicine, where the gene expression analysis was done. Approximately 50–100 mg of liver tissue was homogenized in 1-mL Trizol (TakaRa Biotechnology, Dalian, China). Total RNA was isolated from frozen liver tissue according to the RNA isolation kit (Gentra, Minneapolis, MN, USA) following the manufacturer’s protocols. The purity and integrity of the total RNA were monitored by absorbance of an ultraviolet spectrophotometer at 260/280 nm. For the synthesis of complementary DNA (cDNA), the extracted RNA was reverse-transcribed by QuantiTect SYBR Green RT–PCR kit (Qiagen, Hilden, Germany; catalog No.204243), as recommended by the manufacturer. The primers were designed with Primer3 software and are listed in Table 1. The PCR was performed in 25 mL containing 12.5-mL QuantiFast SYBR Green (catalog No. 204141), PCR Master Mix, 1 mM of each primer (Invitrogen, Karnataka, India), and 2-mL cDNA. For Enho, RAR-related orphan receptor alpha, and Rev-Erb-α, denaturation was done at 95 °C for 5 min, and 40 cycles were performed: annealing at 53 °C and extension at 70 °C for 45 s and denature at 95 °C for 10 s. A dissociation curve was performed after finishing 40 cycles to verify the quality of the primers and amplification. Relative expression of the genes was calculated by the Ct-method and normalized to the housekeeping gene β-actin.

### 2.6. Hormones, Enzymes, and Substrates Measurements

Plasma Adropin levels were measured using enzyme-linked immunosorbent assay (ELISA) kits for rat Adropin (catalog number S-1385; Peninsula Laboratories International, Inc., San Carlos, CA, USA), as described by the manufacturer’s catalog. The key enzymes of the lipid metabolism, such as Hormone-sensitive lipase (HSL) and adipocyte triglyceride lipase (ATGL), were measured in the plasma samples by ELISA Kits, (catalog numbers MBS762158 and MBS2503928, respectively; MyBiosource, San Diego, CF, USA) with the sensitivities of <2 pg/mL and 0.469 ng/mL, respectively. The free fatty acid (FFA) concentration was assessed by kits for the FFA quantification colorimetric assay (catalog number MB S841629; MyBiosource, San Diego, CF, USA).

### 2.7. Statistical Analysis

The data were presented as the means ± standard deviation (SD). Shapiro–Wilk test was used to test the normality of study variables. Analysis of variance (ANOVA) with an LCD post hoc test was used to analyze the differences in multiple comparisons; additionally, the significance in the multiple comparisons were tested by Bonferroni correction. *p*-values < 0.05 were statistically significant. SPSS, version 25 for Windows software (SPSS Inc., Chicago, IL, USA) was used for the statistical analyses.

## 3. Results

### 3.1. Effects of Continuous Light Exposure

**Figure 2:**
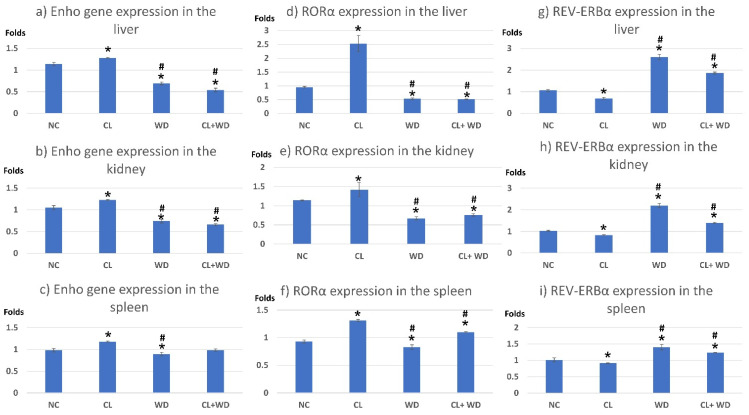
Expression of Enho, RORα, and Rev-erb-α genes in the peripheral tissues. * significant vs. NC group and # significant vs. CL group. (**a**) Enho gene expression in the liver; (**b**) Enho gene expression in the kidney; (**c**) Enho gene expression in the spleen; (**d**) RORα expression in the liver; (**e**) RORα expression in the kidney; (**f**) RORα expression in the spleen; (**g**) REV-ERBα expression in the liver; (**h**) REV-ERBα expression in the kidney; (**i**) REV-ERBα expression in the spleen.

**Figure 3:**
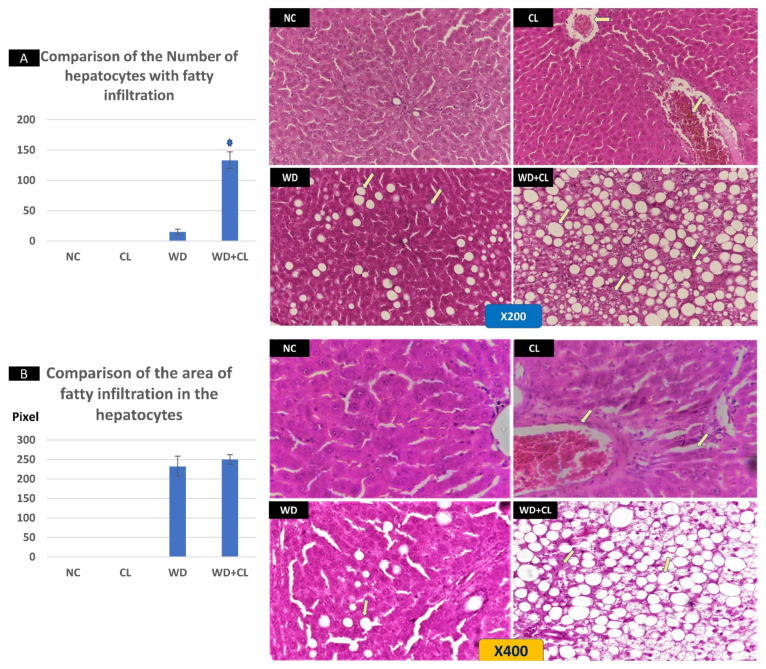
Histopathological changes in the liver tissue in different study groups at ×200 (above) and ×400 (below). The normal control (NC) slide shows normal hepatic tissue. Continuous light (CL) shows some congested hemorrhagic areas of different sizes and shapes (arrows) surrounded by degenerated hepatocytes, while the remaining tissue is normal. Western diet (WD) sections show the early fatty degeneration of hepatocytes with the intracellular deposition of fat droplets (both micro- and macro-vesicular steatosis) (arrows). Combined WD + CL shows that advanced fatty degeneration was noticed in the liver parenchyma with few remnants of compressed hepatocytes (arrows). The number of hepatocytes with macro-vesicular steatosis is significantly higher in the WD + CL (**A**). However, the size of hepatocytes with macro-vesicular steatosis is relatively similar in both WD and WD + CL (**B**). * *p* < 0.05.

**Figure 4:**
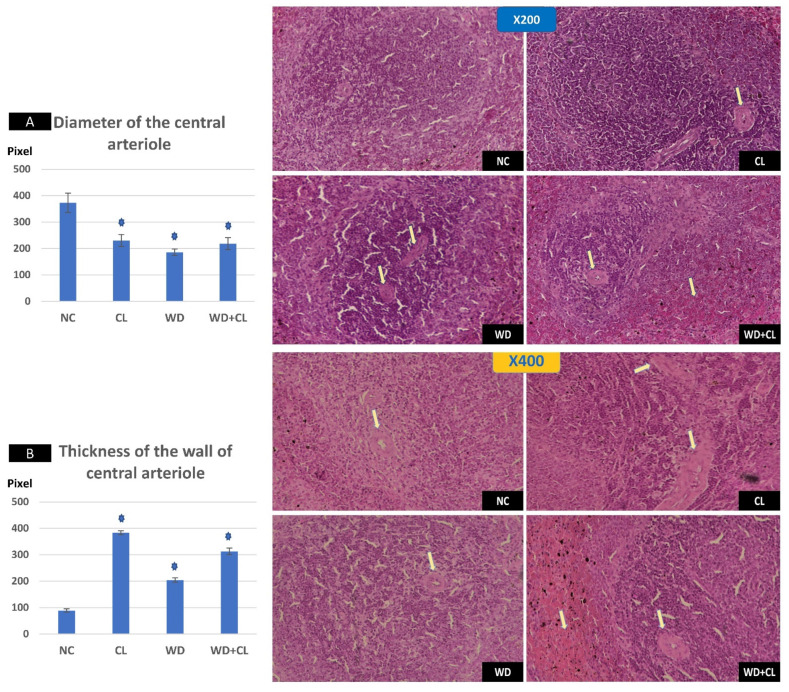
Histopathological changes in the spleen among the study groups at ×200 (above) and ×400 (below). The normal control (NC) section shows normal central arteriole, white pulp, and red pulp. Continuous light (CL) shows a thick-walled and narrow lumened central arteriole (arrow). Western diet (WD) slide shows moderate wall thickening and the narrow lumen of the central arterioles (arrows). Combined WD + CL shows wall thickening and narrow lumen of the central arterioles splenic tissue areas with congestion and hemorrhaging (arrows). Diameter of the central arteriole (**A**) and wall thickness (**B**) were significantly different than the NC group. * *p* < 0.05.

**Figure 5:**
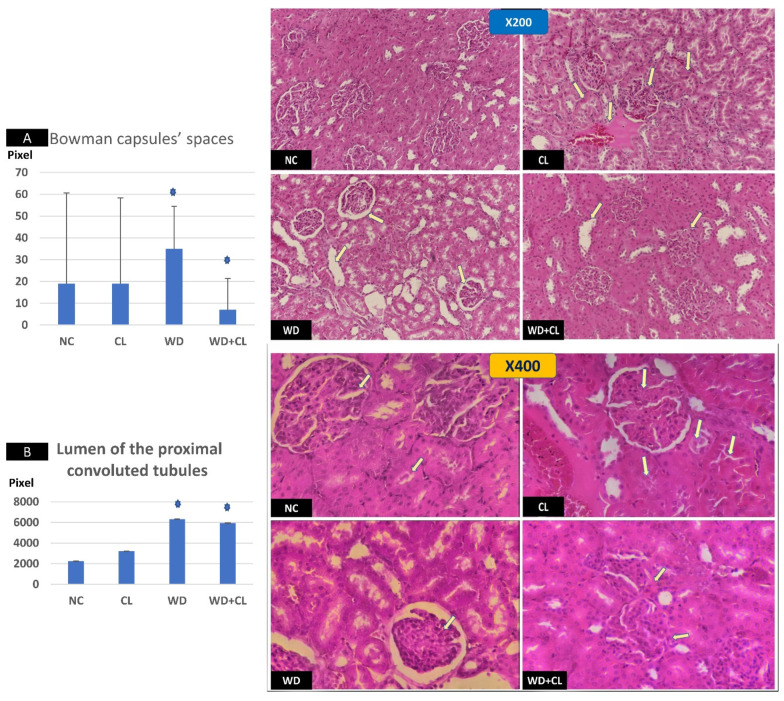
Sections in the renal cortex at ×200 (above) and ×400 (below). The normal control (NC) section shows normal glomeruli. Continuous light (CL) shows a thick glomerular membrane with the cloudy swelling of proximal convoluted tubules (PCT) in form of the increased size of lining epithelium, cloudy cytoplasm, and star-shaped lumen (arrows). Western diet (WD) slide shows inflammatory and hypertrophic changes in the glomeruli with increased Bowman capsular space (arrows). Combined WD + CL shows the disappearance of Bowman capsule spaces with mild inflammatory cell infiltration (arrows). Image J tool measurements showed a significant dilatation of Bowman capsule spaces in the WD group and significant narrowing in the WD + CL (**A**). The lumen of the PCT showed progressive dilatation, especially the WD and WD + CL groups (**B**). * *p* < 0.05.

**Figure 6:**
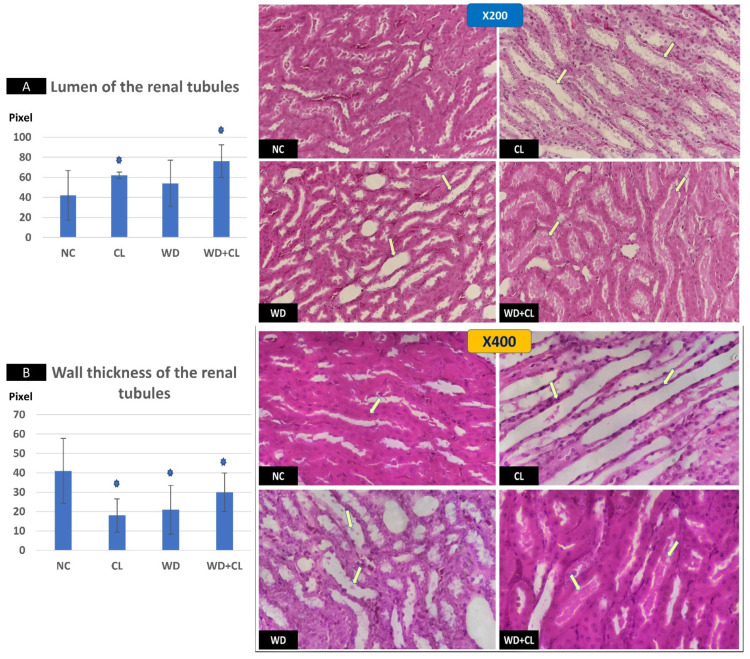
Sections in renal medulla showing renal tubules in different study groups at ×200 (above) and ×400 (below). The normal control (NC) section shows normal tubules. Continuous light (CL) shows dilated elongated collecting ducts filled with clear material (arrows). Western diet (WD) slide tubules were dilated with sloughed epithelium into the lumen (arrows). Combined WD + CL shows moderate dilatation with the presence of homogenous pink casts in the lumen (arrows). Compared to the NC group, the lumen of the renal tubules was dilated (**A**), and the wall-thickness was decreased (**B**). * *p* < 0.05.

Compared to the NC group, prolonged light exposure alone elevated the Adropin expression and circulating plasma level (33.04 ± 2.34 vs. 21.39 ± 1.17 ng/mL, *p* < 0.001) (Table 2). The peak Enho gene expression in the liver, kidney, and spleen coincided with increased RORα expression and decreased Rev-erb-α nuclear receptor expression, mainly in the liver and kidney (Figure 2). Besides, CL increased the energy expenditure (TEE), decreased the respiratory quotient (RQ) toward the fat oxidation zone, and decreased the food intake, producing an insignificant reduction of weight gain (*p* = 0.173) (Table 3). Compared to the NC group, the water intake was insignificantly changed (*p* > 0.05). Although both HSL and ATGL were insignificantly changed (*p* > 0.05), the level of the FFA was significantly reduced (might be cleared for oxidation). In comparison to the NC group, a histopathology examination of the liver showed congested hemorrhagic areas of different sizes and surrounded by degenerated hepatocytes (Figure 3CL; ×200 and ×400). As showed in Figure 4CL, a thick-walled and narrow, lumened central arteriole due to the deposition of homogenous pink hyaline material in the wall. This finding was demonstrated by the histomorphometric analysis using image J (Figure 4A,B). Sections in the kidney tissue showed thickening of the glomerular membrane with cloudy swelling of proximal convoluted tubules in the form of the increased size of the lining epithelium, cloudy cytoplasm, and star-shaped lumen. Besides, some regions of interest (ROIs) showed focal areas of homogenous eosinophilic material with focal hemorrhage (Figure 5CL). Sections in the renal tubules showed dilated elongated collecting ducts filled with a clear material that might indicate acute tubular necrosis (Figure 6-CL; ×200 and ×400).

### 3.2. Effects of Western Diet Ingestion

Compared to the NC group, a Western diet alone significantly reduced the Adropin gene expression in the studied peripheral tissues and decreased its plasma level. Furthermore, the expression of RORα was parallel to the Enho gene, while Rev-erb-α showed a high expression (Figure 2). Changes in the TEE, RQ, volumes of respiratory gases, and water intake in the WD group were insignificant, while the food intake was reduced in comparison to the NC group (Table 3). The lipolytic enzymes in the WD group vs. the NC group showed a significant increase in the case of ATGL rather than HSL, leading to a rise in FFA concentration in the plasma (Table 2). Liver parenchyma in the WD group showed early fatty degeneration of hepatocytes with the intracellular deposition of fat droplets (both micro- and macro-vesicular steatosis) (Figure 3WD; ×200 and ×400). Compared to the NC group, splenic ROI showed moderate wall thickening and narrow lumen of the central arterioles (Figure 4WD). In the kidney, some inflammatory and hypertrophic changes in the glomeruli were detected, while the tubules were dilated with sloughed epithelium into the lumen (Figure 5 and Figure 6WD; ×200 and ×400). High-power examinations (×400) and an image J measurement analysis further proved these findings.

### 3.3. Effects of Western Diet Added to Continuous Light Exposure

Adding the CL to WD did not increase Enho gene expression in the peripheral tissues and plasma level, indicating that the effect of diet is more potent than light exposure. This was the case also in the expression of the RORα and Rev-erb-α (Figure 2). Compared to the NC, combined CL and WD further increased the TEE and lowered the RQ (i.e., the main substrate used was the fat) and reduced the food intake, reducing the weight gain near-significantly (*p*-value of the post hoc test = 0.058) (Table 3). Furthermore, the increased ATGL was maintained significantly higher than the conditions in the CL alone and NC groups. Microscopically, widespread advanced fatty degeneration was noticed in the liver parenchyma with a few remnants of compressed hepatocytes (Figure 3WD + CL; ×200 and ×400). In addition to wall thickening and the narrow lumen of the central arterioles, the splenic tissue showed areas with congestion and hemorrhage (Figure 4WD + CL; ×200 and ×400). The selected ROI from the kidney showed the disappearance of Bowman capsule spaces with mild inflammatory cell infiltration. Additionally, renal tubules showed moderate dilatation with the presence of homogenous pink casts in the lumen (Figure 5 and Figure 6WD + CL; ×200 and ×400).

### 3.4. Circadian Rhythms of Plasma Adropin

**Figure 7:**
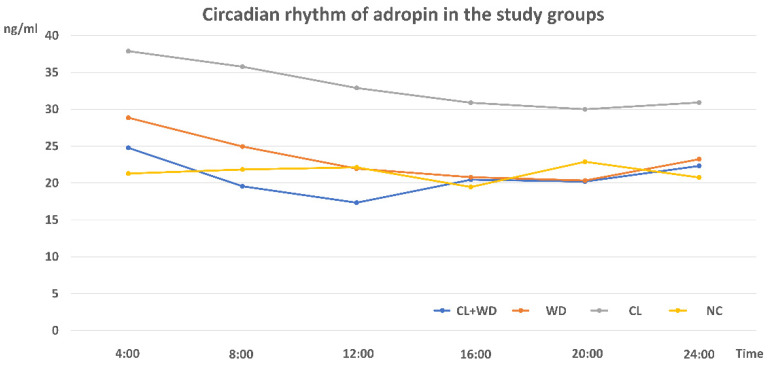
Circadian rhythms of plasma Adropin in different study groups (*n* = 3/group).

The patterns of the peripheral circadian changes in plasma Adropin were presented in Figure 7. In the NC group, there is no significant variations among the tested time points; however, some fluctuation was observed at the dark period. These fluctuations were masked by both CL and WD, with relatively higher levels in the early morning period than in the evening. Combined WD and CL made the rhythm like that of the WD with a persistent loss of night peaks.

## 4. Discussion

The current study investigated the individual and combined effects of two factors related to the modern lifestyle on metabolic health. The first factor was the extended exposure of light at the dark phase of the normal light/dark cycle (CL), and the second was the ingestion of foods with a high-fat proportion and added sugars containing beverages (WD). The effects on energy homeostasis, lipolysis key enzymes, Adropin peptide, and related nuclear receptors will be discussed here.

In the CL group, Adropin expressions in peripheral tissues, i.e., liver, spleen, and kidney, were increased, and the plasma level was elevated. This effect was associated with a significant reduction of the free fatty acid level in the plasma, with insignificant changes in the lipolysis key enzymes (HSL and ATGL), meaning that Adropin may have an independent antilipolytic effect. In-line with this assumption, Gao et al. [25] stated that Adropin overexpression or the administration of the Adropin agonist can suppress lipolysis, enhance glycolysis, and improve the glucose tolerance. The overexpression of the Enho gene coincided with the overexpression of RORα and under-expression of Rev-erb-α in the studied peripheral tissues. Indicating that RORα and Rev-erb-α may play a molecular mechanism for Adropin action. Previous reports indicated that ROR antagonists could suppress the Enho gene expression in human cell cultures [14]. Furthermore, the nocturnal peak of Adropin secretion was associated with activation of the RORα and RORγ factors [12].

Besides Adropin, the sympathetic nervous system (SNS) is also disturbed by extended periods of light exposure. Light/dark cycle alterations could modify the norepinephrine (NE) level in tissues innervated by SNS directly by actions on the rostral ventrolateral medulla, superior cervical sympathetic ganglia, and alterations in the hormonal release, resulting in sympathetic hyperactivity [26]. The hyperactivity of SNS may explain the significant reduction of the RQ toward the fat utilization zone, increased TEE, reduction of food intake, and reduction of weight gain in the CL group. Adipose tissue is innervated by the SNS, and lipolysis in the white adipose tissue is induced by NE-mediated SNS activation [27]. In humans, the CL may produce a different effect with an increased tendency to obesity due to increased food intake and disturbed meal timing. This may be evident in night eaters [28] and individuals with a delayed hour of going to sleep [29]. Physical inactivity is an important factor affecting this contrary finding in human studies. It was reported that access to a voluntary exercise by a running wheel for experimental animals prevented weight gain in mice exposed to light at night [30]. A third opinion was also present in human studies where Melanson et al. [31] stated that continuous bright light exposure even at night had little to no effect on TEE by direct calorimetry, hourly RQ, and glucose metabolism in normal young adults.

Furthermore, CL could disturb other modulators of the metabolism, such as melatonin [32] and glucocorticoids [33]. The inhibition of melatonin by light contamination of the dark phase is evident in both human and experimental animals. Inhibited nocturnal melatonin release was associated with an increased risk of type 2 diabetes and obesity in human studies [3]. Regarding glucocorticoids, CL may increase, decrease, or not affect its concentrations, meaning that CL-induced changes in glucocorticoids are not critical for the metabolic disturbances associated with nocturnal light exposure [34].

The deleterious effect of the CL on the liver, spleen, and renal histology could support the theory of SNS overactivity (i.e., Cl is a stressful stimulus). The widespread degenerative changes in the studied tissues were like pathological findings in stress models, which were created in experimental animals by the chronic administration of epinephrine for four weeks [35] or foot shock for three weeks [36].

Rats fed a high-fat diet with added sucrose (in the WD group) for 12 weeks failed to produce a significant weight gain. Going through the indirect calorimetry study, it was evident that the TEE and RQ were insignificantly changed when compared to the NC group. Moreover, the automatic measurement of the food intake revealed a significant reduction in the food intake in the WD group. In contrary to these findings, Collins et al. [19] found that a high-fat/sucrose diet (HFS) for one week increased the fat mass without weight gain, while for four weeks, it produced fat and weight gains. The longer duration in our study could give an understanding of this discrepancy. The longer duration could produce enough time for the development of an insulin resistance, which was indicated in the current study by increased ATGL and FFA levels in the WD group. It is reported that the imbalance between HSL and ATGL affects diacylglycerol (DAG) accumulation in skeletal muscle and predispose to insulin resistance [37]. Additionally, high FFA increases protein kinase C (PKC) activation, resulting in the inhibition of insulin signaling pathways. The HFS diet leads to atrophy of the skeletal muscles, especially the myofibrillar proteins, leading to a loss of muscle mass [38]. Additionally, during a long duration, rats may adapt to HFS feeding and become resistant to gain weight and/or other metabolic disturbances [39].

Rats in the WD group showed significantly reduced the Adropin gene expression and plasma level together with the under-expression of RORα and overexpression of Rev-erb-α. Many previous reports associated the reduction of Adropin levels with insulin resistance conditions. A low Adropin level can be considered a risk factor for insulin resistance and other components of the metabolic syndrome [40]. Additionally, Adropin can regulate the expression of the lipogenic genes in the liver and adipose tissue peroxisome proliferator-activated receptor-γ (regulator of lipogenesis); therefore, it may behave as a factor controlling glucose and lipid homeostasis, which protects against hepatosteatosis and insulin resistance [41].

The theory of insulin resistance was clear in the histopathological changes in the liver parenchyma, where a picture like nonalcoholic fatty liver disease (NAFLD) was evident with early to advanced widespread steatosis in all studied slides of the WD group. It was reported that a low serum level of Adropin was associated with NAFLD in adult patients. The hypoadropinemia was linked to the NAFLD pathogenesis through Adropin-related endothelial dysfunction, depending on the ability of Adropin to induce the expression of nitric oxide (NO) into the endothelium, i.e., hypoadropinemia will be associated with a reduction of the NO in endothelial cells [42]. Sterol regulatory element-binding protein (SREBP) 1c is an important transcription factor implicated in the pathogenesis of NAFLD [43]. Interestingly, Rev-erb-α could participate in the circadian regulation of SREBP1c activity and expression of SREBP-targeted genes linked to cholesterol and lipid metabolism [44]. The changes in the splenic central arteriole, glomerular inflammation, and hypertrophy, and renal tubular changes might be understood in the context of inflammatory changes associated with insulin resistance and hypoadropinemia. Li et al. [45] reported a reduction of the antioxidant capacity of kidney tissues after long-term high-fat high-sucrose feeding in Bama Minipigs in addition to chronic renal tissue injury similar in part to our findings.

The combined effect of CL and WD indicated that the WD effect on Adropin is more potent than CL, i.e., Adropin level and expression together with expressions of RORα and Rev-erb-α were like the case in the WD. On the other hand, the stressing effect of adding CL to WD (state of insulin resistance) was synergistic regarding the indirect calorimetry findings (increased TEE, and lowered RQ and reduction in the weight gain) and degenerative changes in the studied tissues. Regarding the energy homeostasis, long-term combined CL and WD further increased the TEE and lowered RQ (toward fat utilization) and reduced the food intake, blunting the weight gain. The finding was corresponding to a previous study showing that CL immediately increased the weight gain then stabilized or even reduced it later due to loss of the central circadian rhythm of energy expenditure and feeding as a result of low amplitude firing of the SCN, whereas the weight gaining effect by the traditional high-fat diet became evident at a later stage than that by the CL, indicating that the CL has an independent mechanism of wight affection [46].

The plasma Adropin circadian rhythm in the NC showed small peaks at night that might be attributed to the time of feeding [14]. On the other hand, Butler et al. [47] stated that ENHO gene in silico expression showed a dynamic and diurnal pattern. Plasma Adropin rhythm was reversed in the CL and WD groups with blunting of the fluctuations and showing higher levels at daytime rather than nighttime. However, the means of the Adropin levels were much lower in the WD group. The combined CL plus WD showing a pattern like WD is an indication that WD is a more potent effector on the Adropin level than CL. Notably, the Adropin plasma level and expression were tested in all study groups after overnight fasting. Despite being a common practice, it was to minimize the stimulatory effects of feeding.

The current study encountered some limitations, such as a lack of measurement of the other indicators of insulin resistance. Alternatively, we depended on the FFA level and dysregulated HSL/ATGL balance as indicators of insulin resistance. Additionally, we measured the TEE rather than the resting energy expenditure. However, the effect of the TEE in energy homeostasis is the best. Another limitation is the lack of measurement of stress indicators or signs of SNS activation. During the study design, it was not clear that the CL will behave as a stress stimulus, and our objective was directed mainly toward Adropin and its associated nuclear factors. Additionally, the comparison with brain and adipose tissue parameters was missing.

## 5. Conclusions

In conclusion, the widespread deleterious effects of the CL and WD on the energy homeostasis and lipid metabolism may include the Adropin peptide with the involvement of the RORα and Rev-erb-α nuclear receptors as a mechanism. The effect of WD was more potent than CL regarding the Adropin expression in peripheral tissues and the plasma level, while the degenerative histological changes showed a synergistic effect of both CL as a stressful factor and WD as an insulin resistance condition.

## Figures and Tables

**Table 1:** Primer (Invitrogen, USA) sequences for the Enho, RAR-related orphan receptor alpha, Rev-Erb- α, and B-actine genes.

Reverse Primer	Forward Primer	Gene
R: 5TGGCTGTCCTGTCCACACAC 3′	F: 5′ ACCGGGCTCAACTCAGGC 3′	Enho (Adropin)
R: 5′GAGCGATCCGCTGACATCA 3′	F: 5′ GCACCTGACCGAAGACGAAA 3′	RAR-related orphan receptor alpha
R: 5′ TGCCATTGGAGCTGTCACTGTAG 3′	F: 5′ GTGAAGACATGACGACCCTGGA 3′	Rev-erb-α
R: 5′ GGGCAACATAGCACAGCTTCT 3′	F: 5′ TGACCGAGCGTGGCTACAG 3′	B-actine

**Table 2:** Weight changes and lipid metabolism parameters among the studied groups (*n* = 8 per each group).

Variables	NC Group Mean ± SD (*n* = 8)	CL Group Mean ± SD (*n* = 8)	WD Group Mean ± SD (*n* = 8)	CL + WD Group Mean ± SD (*n* = 8)	*p*-Value
Basal Weight (g)	256.17 ± 7.88 ^a^	265.00 ± 27.07 ^a^	262.00 ± 22.24 ^a^	267.17 ± 25.27 ^a^	0.837
Final Weight (g)	360.00 ± 15.8 ^a^	343.83 ± 44.70 ^a^	357.00 ± 22.70 ^a^	335.33 ± 22.78 ^b^	0.426
Adropin Level (ng/mL)	21.39 ± 1.17 ^a^	33.04 ± 2.34 ^b^	18.81 ± 1.86 ^b^	17.67 ± 2.04 ^b^	0.000
HSL Level (pg/mL)	112.59 ± 9.57 ^a^	120.60 ± 6.92 ^a^	106.62 ± 13.5 ^a^	110.40 ± 12.42 ^a^	0.188
ATGL (ng/mL)	11.14 ± 1.62 ^a^	12.39 ± 2.20 ^a^	40.67 ± 1.85 ^b^	41.83 ± 1.56 ^b^	0.000
FFA Level (ng/dL)	562.12 ± 16.08 ^a^	490.51 ± 7.80 ^b^	654.04 ± 64.9 ^b^	439.68 ± 2.55 ^b^	0.000

Values with the different superscripts within a raw are statistically significant according to the LCD post hoc test with Bonferroni correction (*p* < 0.05). NC = normal control, CL = Continuous light group, WD = western diet group, HSL = hormone-sensitive lipase, ATGL= adipocyte triglycerides lipase, and FFA = free fatty acids.

**Table 3:** Indirect calorimetry parameters of all studied groups (*n* = 8/group).

Variables	NC Group Mean ± SD (*n* = 8)	CL Group Mean ± SD (*n* = 8)	WD Group Mean ± SD (*n* = 8)	CL + WD Group Mean ± SD (*n* = 8)	*p*-Value
VO_2_ (ML/H/kg)	1256.23 ± 112.52 ^a^	1397.65 ± 140.47 ^b^	1189.34 ± 52.58 ^a^	1454.18 ± 115.05 ^b^	0.002
VO_2_ (ML/H/kg LBM)	948.41 ± 83.40 ^a^	1065.50 ± 79.04 ^b^	919.55 ± 29.96 ^a^	1109.80 ± 78.75 ^b^	0.000
VO_2_ (ML/H/RAT)	409.16 ± 47.38 ^a^	474.60 ± 32.95 ^b^	425.54 ± 17.38 ^a^	494.56 ± 41.68 ^b^	0.002
VCO_2_ (ML/H/kg)	1112.74 ± 175.68 ^a^	1096.95 ± 140.75 ^a^	1009.06 ± 48.27 ^a^	979.59 ± 47.60 ^a^	0.231
VCO_2_ (ML/H/kg LBM)	840.16 ± 131.32 ^a^	815.99 ± 94.50 ^a^	780.21 ± 30.64 ^a^	747.87 ± 33.35 ^a^	0.274
VCO_2_ (ML/H/RAT)	362.60 ± 62.85 ^a^	363.89 ± 45.32 ^a^	361.15 ± 18.55 ^a^	333.71 ± 28.61 ^a^	0.563
RQ	0.88 ± 0.07 ^a^	0.76 ± 0.05 ^b^	0.85 ± 0.03 ^a^	0.67 ± 0.04 ^b^	0.000
TEE (KCAL/H/kg)	6.18 ± 0.63 ^a^	6.69 ± 0.70 ^a^	5.80 ± 0.25 ^a^	6.81 ± 0.50 ^a^	0.016
TEE (KCAL/H/kg LBM)	4.67 ± 0.47 ^a^	5.10 ± 0.40 ^b^	4.49 ± 0.14 ^a^	5.20 ± 0.34 ^b^	0.007
TEE (KCAL/H/RAT)	2.01 ± 0.25 ^a^	2.27 ± 0.18 ^b^	2.08 ± 0.08 ^a^	2.32 ± 0.19 ^b^	0.029
Food Intake (G/RAT)	49.50 ± 15.18 ^a^	24.17 ± 10.07 ^b^	22.58 ± 4.37 ^b^	15.51 ± 7.94 ^b^	0.000
Water Intake (ML/kg/DAY)	82.00 ± 4.00	84.17 ± 10.42	86.33 ± 7.10	86.2 ± 8.33	0.750
Weight Gain (G)	103.33 ± 21.68 ^a^	78.83 ± 34.02 ^a^	95.00 ± 33.80 ^a^	68.17 ± 31.54 ^a^	0.214

Values with the different superscripts within a raw are statistically significant according to the LCD post hoc test with Bonferroni correction (*p*  <  0.05). VO_2_ = Volume of consumed oxygen, VCO_2_ = volume of the produced carbon dioxide, LBM = lean body mass, RQ = respiratory quotient (VCO2/VO2), and TEE = total energy expenditure.

## Data Availability

The raw data supporting the conclusions of this article will be made available by the authors, without undue reservation, to any qualified researcher.
