# Correction: Abulmeaty et al. Effect of Long-Term Continuous Light Exposure and Western Diet on Adropin Expression, Lipid Metabolism, and Energy Homeostasis in Rats. *Biology* 2021, *10*, 413

**DOI:** 10.3390/biology12020209

**Published:** 2023-01-29

**Authors:** Mahmoud Mustafa Ali Abulmeaty, Ali Madi Almajwal, Khalid S. Alnumair, Suhail Razak, Mai Mohammed Hasan, Amal Fawzy, Abdullah Ibrahim Farraj, Manal Abudawood, Ghadeer S. Aljuraiban

**Affiliations:** 1 Department of Community Health Sciences, College of Applied Medical Sciences, King Saud University, Riyadh 11362, Saudi Arabia; 2 Department of Medical Physiology, School of Medicine, Zagazig University, Zagazig 44519, Egypt; 3 Department of Medical Biochemistry, School of Medicine, Zagazig University, Zagazig 44519, Egypt; 4 Department of Clinical Laboratory Sciences, College of Applied Medical Sciences, King Saud University, Riyadh 11362, Saudi Arabia

In the original publication [1], the funder “The Deputyship for Research & Innovation, Ministry of Education in Saudi Arabia”, “The project no. (IFKSURG-2-940)” to “Mahmoud Mustafa Ali Abulmeaty” was not included. A correction has been made to “Funding” and “Acknowledgment” sections:

Funding: This project was funded by The Deputyship for Research & Innovation, Ministry of Education in Saudi Arabia, the project no. (IFKSURG-2-940).
Acknowledgments: The authors extend their appreciation to the Deputyship for Research & Innovation, Ministry of Education in Saudi Arabia for funding this research work through the project no. (IFKSURG-2-940).

The authors state that the scientific conclusions are unaffected. This correction was approved by the Academic Editor. The original publication has also been updated.
